# Neural Oscillation Profiles of a Premise Monotonicity Effect During Semantic Category-Based Induction

**DOI:** 10.3389/fnhum.2019.00338

**Published:** 2019-10-15

**Authors:** Mingze Sun, Feng Xiao, Changquan Long

**Affiliations:** ^1^Key Laboratory of Cognition and Personality of MOE, Southwest University, Chongqing, China; ^2^Department of Education Science, Innovation Center for Fundamental Education Quality Enhancement of Shanxi Province, Shanxi Normal University, Linfen, China

**Keywords:** category-based induction, non-phase-locked power, phase-locked power, premise monotonicity effect, connectionist models, time-frequency analysis

## Abstract

A premise monotonicity effect during category-based induction is a robust effect, in which participants are more likely to generalize properties shared by many instances rather than those shared by few instances. Previous studies have shown the event-related potentials (ERPs) elicited by this effect. However, the neural oscillations in the brain underlying this effect are not well known, and such oscillations can convey task-related cognitive processing information which is lost in traditional ERP analysis. In the present study, the phase-locked and non-phase-locked power of neural oscillations related to this effect were measured by manipulating the premise sample size [single (S) vs. two (T)] in a semantic category-based induction task. For phase-locked power, the results illustrated that the premise monotonicity effect was revealed by anterior delta power, suggesting differences in working memory updating. The results also illustrated that T arguments evoked larger posterior theta-alpha power than S arguments, suggesting that T arguments led to enhanced subjectively perceived inductive confidence than S arguments. For non-phase-locked power, the results illustrated that the premise monotonicity effect was indicated by anterior theta power, suggesting that the differences in sample size were related to a change in the need for cognitive control and the implementation of adaptive cognitive control. Moreover, the results illustrated that the premise monotonicity effect was revealed by alpha-beta power, which suggested the unification of sentence and inference-driven information. Therefore, the neural oscillation profiles of the premise monotonicity effect during semantic category-based induction were elucidated, and supported the connectionist models of category-based induction.

## Introduction

Category-based induction plays a significant role in human learning and adaptation (Anderson, 1991; Heit and Hayes, 2011), and involves exploiting knowledge about a property of the premise categories to infer the same property about the members of a conclusion category (Kemp and Jern, 2014; Hayes and Heit, 2018). For example, if we know all tigers (premise category) have gene M, we could infer that all mammals (conclusion category) have gene M, since we know that tigers are members of the category of mammals.

One of the typical psychological effects during category-based induction is the premise monotonicity effect. This effect reflects the observation that individuals are more likely to generalize properties shared by many instances than those shared by few instances (Osherson et al., 1990; Feeney, 2007). For instance, it might be easier to generalize that all mammals have gene M based on the premise that both tigers and lions have gene M than based on the premise that only lions have gene M, because the former argument has a larger sample size than the latter. This effect is robust, and can be observed in children (Gutheil and Gelman, 1997; Li et al., 2009; Lawson, 2014; Rhodes and Liebenson, 2015), healthy adults (Osherson et al., 1990; Rotello and Heit, 2009; Feeney and Heit, 2011), and adults with obsessive-compulsive disorder (Liew et al., 2018).

Cui et al. (2018) measured the event-related potentials (ERPs) elicited by the premise monotonicity effect during category-based induction to explore the timing of brain activity underlying this effect. In their study, they manipulated the types of premise categories *via* displaying one (S arguments, e.g., tigers) or two types of premise categories (T arguments, e.g., tigers and lions) with a novel property (e.g., X1) in the arguments. Their results suggested that the cognitive processes underlying the premise monotonicity effect during category-based induction involved familiarity, which produced FN400 (a negative deflection with frontal-central distribution at 250–450 ms), and inference-driven information integration, which produced sustained negativity (SN, a prolonged negative deflection that lacks a clear peak and features a similar latency window as that of the FN400 effect).

However, some task-related cognitive processes underlying the premise monotonicity effect during category-based induction would be lost in the ERP study of Cui et al. (2018), due to the limitations of traditional ERP techniques. ERPs reflect the summation of the postsynaptic potentials involving a large ensemble of active neurons (Cohen, 2014; Cavanagh, 2019). In traditional ERP analysis, time-locked and phase-locked activity are averaged as event-related EEG signals, while the time-locked and non-phase-locked activity, which provide important information related to cognitive processing, are lost (Cohen, 2014). Thus, some task-related cognitive processes underlying the premise monotonicity effect during category-based induction would be not revealed in the ERP study of Cui et al. (2018).

The present study aims to reveal the additional cognitive processes underlying the premise monotonicity effect during category-based induction by extending the ERP-based results through the measurement of neural oscillation. Neural oscillations are decomposed using the time-frequency analysis technique, which can identify changes in the amplitude or in the power of the responses within different frequency bands in both phase-locked and non-phase-locked activity, to reveal the cognitive processes that are lost to traditional ERP analysis (Davidson and Indefrey, 2007; Cohen, 2014). The experiment uses the same design and materials, and a similar procedure as Cui et al. (2018). Notably, Liang et al. (2010) performed time-frequency analysis during category-based induction and found that inductive decisions on congruent induction tasks elicited a marginally larger gamma band (30–50 Hz) power than that of incongruent induction tasks. However, they did not explore the neural oscillation profiles of the premise monotonicity effect during semantic category-based induction.

We predicted that the premise monotonicity effect during category-based induction would be revealed by both phase-locked and non-phase-locked EEG power. According to the connectionist models of semantic cognition (Rogers and McClelland, 2008, 2014), cognitive processing units during category-based induction are organized in hierarchical layers, which can include stimuli-driven and experience-based layers. Stimuli-driven bottom-up processing would produce phase-locked EEG power, while experience-based top-down processing would produce non-phase-locked EEG power (Chen et al., 2012; Mei et al., 2018). Thus, we predicted that stimuli-driven cognitive processes would produce phase-locked EEG power, and experience-based cognitive processes would produce non-phase-locked EEG power.

For phase-locked power specifically, we predicted that the premise monotonicity effect during semantic category-based induction would lead to changes in delta and theta-alpha band power, indexing working memory (WM) updating, subjectively perceived confidence, and cognitive processing facilitation. In the present study, more WM needs to be updated in T arguments with incongruent conclusion (T−) than in S arguments with incongruent conclusion (S−), because of the increased conceptual confliction during T−; and less WM needs to be updated in T arguments with congruent conclusion (T+) than in S arguments with congruent conclusion (S+), because of the increased conceptual fluency. WM updating is related to increased delta power (Harmony, 2013; Rac-Lubashevsky and Kessler, 2018), and thus we predicted that T− would evoke larger delta power than S−, while T+ would evoke smaller delta power than S+. In addition, cognitive processing would be facilitated by inhibiting stimulus-based bottom-up processing demands during T arguments compared with S arguments, thus producing increased subjectively perceived confidence. The facilitation of the inhibition on stimulus-based bottom-up processing demands and subjectively perceived confidence were associated with larger posterior alpha (e.g., Klimesch, 2012) and posterior theta power (Wynn et al., 2019), respectively. We thus predicted that T arguments would have larger evoked posterior theta-alpha band power than S arguments.

For non-phase-locked power, we predicted that the premise monotonicity effect during semantic category-based induction would lead to changes in theta and alpha-beta power, indexing cognitive control and unification respectively. In the present study, T− is characterized by stronger conflict than S− because of the double categorical membership conflict, and S arguments involve more adaptive control because of the increased response caution compared with T arguments. These processes involve distinctive requirements for cognitive control or adaptive control. Anterior theta power is related to cognitive control (Cavanagh and Frank, 2014; Cavanagh and Shackman, 2015; Helfrich et al., 2019). We, therefore, predicted that the premise monotonicity effect during semantic category-based induction could be revealed by anterior theta power in a relatively early time window for cognitive control requirements, and in a relatively late time window for adaptive control. In addition, T arguments need more complicated sentence integration than S arguments, whereas they produce less inference-driven information unification than S arguments due to the larger sample size providing more evidence. The increased information unification is related to decreased alpha-beta band power (Hagoort, 2005, 2013; Lam et al., 2016). We, therefore, predicted that T arguments would generate lower (larger) alpha-beta power than S arguments in a relatively early (late) period.

## Materials and Methods

### Ethical Statements

This study was approved by the ethics review board of the Faculty of Psychology, Southwest University, Chongqing, China. Written informed consent was obtained from all participants. All procedures involved were performed in accordance with the Declaration of Helsinki (World Medical Association, 2013).

### Participants

The participants in the present experiment do not overlap those of the study by Cui et al. (2018). Forty-two undergraduate students [mean (*M*) age: 20.21 years; standard deviation (*SD*): 1.44; range: 18–24 years, 28 females] were recruited. All participants were self-reported as right-handed, native Mandarin Chinese speakers, had a normal or corrected-to-normal vision, and no neurological impairment.

### Experimental Materials, Design, and Procedure

The experimental materials and design are the same as in the study of Cui et al. (2018), to which we refer for more detailed information. Briefly, in the present study, to explore the premise monotonicity effect during semantic category-based induction, the experiment was conducted with two premise conditions [single premise category argument (S), two premise category arguments (T)] and two conclusion conditions [congruent (+) or incongruent conclusion (−)] as within-subject factors. The properties used in the present study are a series of molecular structures (e.g., E5, X1). In the formal experiment, each sub-condition included 50 trials. The experimental procedure is similar to the study of Cui et al. (2018), except that the random duration of the blank screen before the conclusion was 1,000–1,200 ms.

Each trial started with a 500 ms black fixation cross (“+”) at the center of the screen. After that, the premises appeared in a random order. In each S argument trial, a single premise was displayed for 800 ms, while in T argument trials a pair of premises (each lasting 800 ms) appeared consecutively, with an 800–1,000 ms random length interval after the first premise. Following a blank screen lasting randomly from 1,000 to 1,200 ms, the conclusion was displayed. Once the conclusion appeared, participants were required to infer the inductive strength of the conclusion based on the premise(s), by choosing one of four degrees (“definitely weak”, “possibly weak”, “possibly strong”, and “definitely strong”). The conclusion disappeared after participants made a response or after 2,000 ms had elapsed. After a blank screen lasting randomly 1,500–2,000 ms, the “+” signal was presented to start a new trial. The experimental trials were divided into four blocks. Sixty seconds of rest was allowed between two consecutive blocks to avoid fatigue effects.

### EEG Recording

Continuous electrophysiological (EEG) signals were recorded *via* an electrode cap (Neuroscan, Herndon, VA, USA), with 64 Ag/AgCl scalp sites according to the International 10/20 system. The ground electrode was placed between FPz and Fz. The online reference electrode was located between Cz and CPz. The vertical electrooculograms (EOGs) were recorded supra-orbitally and infra-orbitally relative to the left eye; and the horizontal EOG was recorded as the difference in activity of the right vs. the left orbital rim. The impedance of all electrodes was kept below 5 KΩ. The EEG and EOGs were amplified by a SynAmps2 amplifier (Neuroscan) and digitized at a sampling rate of 500 Hz. The signals were recorded in DC mode and amplified with a low-pass filter at 200 Hz with no high-pass filters applied.

### Data Analysis

#### EEG Data Pre-processing

EEG data were analyzed in MATLAB 2014b using the EEGLAB toolbox (Delorme and Makeig, 2004) and the ERPLAB toolbox (Lopez-Calderon and Luck, 2014). EEG data were filtered using second-order IIR-Butterworth filters with 1–50 Hz (half-power cut-offs, roll-off = 12 dB/oct) band pass. A 50 Hz notch filter was also used. Independent component analysis (ICA) was subsequently performed to correct for components associated with eye movements and eye-blinks. The ICA-corrected EEG data were then re-referenced to the average of the left and right mastoid electrodes (Luck, 2014), and segmented into epochs. Among the responses to congruent conclusions, “definitely strong” and “possibly strong” were identified as “correct” responses; “definitely weak” and “possibly weak” responses were identified as “correct” for incongruent conclusions. Only the “correct” responses were selected and segmented into 3,000 ms epochs, including the 1,000 ms preceding the onset of conclusion. The baseline correction was based on the pre-stimulus time interval (−1,000 to 0 ms). Noisy trials were excluded using the moving window peak-to-peak amplitude method (Luck, 2014) with a window width of 200 ms, window step of 100 ms, and a 65-μV threshold. The mean number of trials for each of the four conditions was 42.98 (*SD*: 3.90) for S+, 44.93 (*SD*: 3.65) for T+, 44.55 (*SD*: 3.91) for S−, and 45.60 (*SD*: 3.49) for T−.

#### Time-Domain Analysis

In ERP analysis, the single-trial data were averaged separately for each participant and each condition. The single-participant average waveforms were averaged to obtain group-level average waveforms. According to Cui et al. (2018), FN400 and SN have anterior scalp distribution, and thus the F3, F1, Fz, F2, F4, FC3, FC1, FCz, FC2, and FC4 electrodes were selected and collapsed by averaging their values to give an indicator of anterior activity. Based on the results of Cui et al. (2018), the mean FN400 amplitude was measured during the 250–450 ms time window, and the mean SN amplitude during the 450–1,050 ms time window after the onset of the conclusions.

#### Time-Frequency-Domain Analysis

In time-frequency-domain analysis, the total EEG power was analyzed through the following steps. First, single-trial data were used to estimate the oscillatory power *via* the Morlet continuous wavelet transform (MCWT, Mouraux and Iannetti, 2008). The parameters of central frequency (ω) and restriction (σ) in MCWT were 5 and 0.15, respectively (Tang et al., 2013, 2015). Time-frequency representations (TFRs) were explored in the range of 1–50 Hz in steps of 0.5 Hz. Second, single-trial TFRs were averaged to obtain averaged TFRs of every participant under each condition. Third, the averaged TFRs were subsequently cut in length (−600 to 1,200 ms) to reduce the edge effects. Fourth, the power was normalized by conversion to a decibel (dB) scale [10 * log10 (power/baseline)]. The baseline power was computed as the average power across all experiment conditions, from 600 to 100 ms prior to the onset of the conclusions.

The total EEG power was decomposed into phase-locked and non-phase-locked components. Following Cohen and Donner (2013) and Cohen (2014), under each condition, the non-phase-locked power was obtained by subtracting the ERP from the time-domain EEG signals on each trial and then performing time-frequency decomposition as described above. The phase-locked power was computed by subtracting the non-phase-locked power from the total power.

After obtaining the non-phase-locked and phase-locked power of each condition, to increase statistical strength and reduce false effects (Luck and Gaspelin, 2017), we first identified the spatial regions of interest (S-ROIs): the F3 F1, Fz, F2, F4, FC3, FC1, FCz, FC2, and FC4 electrodes were selected and collapsed by averaging their values to obtain an indicator of anterior activity; the CP3, CP1, CPz, CP2, CP4, P3, P1, Pz, P2, and P4 electrodes were selected and collapsed by averaging their values as an indicator of posterior activity.

Then we performed an exploratory data-driven analysis routine to identify the time-frequency regions of interest (TF-ROIs), based on previous studies (Tang et al., 2013; Tan et al., 2014), with the following steps:

1. Based on the defined S-ROIs, we calculated the difference in magnitude between S arguments and T arguments in both the congruent and incongruent conditions, and that between incongruent and congruent conditions in both S and T arguments, to evaluate the potential effects of premise monotonicity and conclusion congruency.
2. For each time-frequency representation of the (non-)phase-locked magnitude difference, we tested whether and when the resulting (non-)phase-locked magnitudes in the post-stimulus interval were significantly different from the corresponding magnitudes in the pre-stimulus interval using a bootstrapping method (Delorme and Makeig, 2004; Durka et al., 2004).
3. At each time-frequency point, the post-stimulus interval was defined as the investigated population and the interval 600–100 ms before the stimulus was defined as the reference population. The null hypothesis was that there was no difference in mean between these two populations. The pseudo-t-statistic between the two populations was calculated, and its probability distribution was estimated by sampling with replacement two populations of the same size from the reference population. After the permutation was executed 5,000 times, the distribution of the pseudo-t-statistic from the reference population and the bootstrap *p*-values for the null hypothesis were generated.
4. This procedure yielded time-frequency distributions in which the brain responses within the post-stimulus interval were significantly different from the responses in the reference interval (Hu et al., 2012; Peng et al., 2012). To address the problem of multiple comparisons, the significance level (*p*-value) was corrected using a false discovery rate (FDR) procedure (Benjamini and Hochberg, 1995; Benjamini and Yekutieli, 2001). In addition, to control for false-positive observations, significant TF-ROIs were defined based on the following three criteria: (1) the time-frequency pixels were significantly different from the pre-stimulus interval at *p* < 0.01; (2) the time-frequency pixels had to cover more than two nearby significant frequency bands; and (3) the time-frequency pixels had to include more than 125 consecutive significant time points (250-ms; see Hu et al., 2013).
5. After TF-ROIs and S-ROIs were identified, we calculated the mean magnitude within the TF-ROIs at the corresponding S-ROIs for each condition.

In summary, in phase-locked power, two TF-ROIs were identified: delta band (2.5–4 Hz, 100–350 ms); and theta-alpha band (4–12 Hz, 100–500 ms). In non-phase-locked power, four TF-ROIs were identified: early theta band (4–7 Hz, 250–650 ms); late theta band (4–7 Hz, 650–1,100 ms); early alpha-beta band (10–30 Hz, 0–250 ms); and late alpha-beta band (10–30 Hz, 650–1,100 ms).

### Statistical Analysis

For behavioral data, three separate two-factors repeated-measures analysis of variances (ANOVAs) with premise monotonicity (S, T) and conclusion congruency (+,−) as within-subject factors were performed, respectively to analyze response strength, “correct” response rates, and reaction times. Regarding response strength, “definitely weak” was assigned a score of 1; “possibly weak”, 2; “possibly strong”, 3; and “definitely strong”, 4. To analyze the decision threshold between S and T arguments, paired-sample *t*-tests were performed on parameter *c*. Parameter *c* is based on signal detection theory (SDT, MacMillan and Creelman, 2005), in which the “correct” responses under the congruent conclusion conditions were defined as hits, and the “correct” responses under the incongruent conclusion conditions were defined as correct rejections.

For ERP data, two separate two-factors repeated-measures ANOVAs were performed, with premise monotonicity (S, T) and conclusion congruency (+,−) as within-subject factors, to analyze the mean amplitudes of FN400 and SN. For time-frequency domain data, six separate three-factor repeated-measures ANOVAs were performed, with premise monotonicity (S, T), conclusion congruency (+,−), and brain region (anterior, posterior) as within-subject factors. The mean power of the delta and theta-alpha bands in phase-locked power and of the early and late theta and alpha-beta bands in non-phase-locked power were analyzed, respectively.

## Results

### Behavioral Results

Table 1 shows the results of the two-way repeated-measures ANOVAs on response strength, “correct” response rates, reaction times, FN400 amplitudes, and SN amplitudes. For response strength, the interaction between premise monotonicity and conclusion congruency was significant (Table 1). When the factor of premise monotonicity was entered into the *post hoc* analysis, the results suggested that T arguments produced more “definitely” strong responses than S arguments under congruent conditions (T+: *M* = 3.78, *SD* = 0.27; S+: *M* = 3.25, *SD* = 0.44; *F*_(1,41)_ = 56.50, *p* < 0.001, $\eta_{\text{p}}^{2}$ = 0.58), while T arguments produced more “definitely” weak responses than S arguments under incongruent conditions (T−: *M* = 1.12, *SD* = 0.22; S−: *M* = 1.44, *SD* = 0.41; *F*_(1,41)_ = 27.25, *p* < 0.001, $\eta_{\text{p}}^{2}$ = 0.40).

**Table 1:** Results of the two-way repeated-measures ANOVAs of the response strength, “correct” response rate, reaction times, FN400 amplitudes and sustained negativity (SN) amplitudes in anterior region.

		Premise (P)	Conclusion (C)	C*P
Response strength	*F*_(1,41)_	13.52	957.99	49.948
	*P*	0.001	<0.001	<0.001
	$\eta_{\text{p}}^{2}$	0.248	0.959	0.549
“Correct” response rate	*F*_(1,41)_	44.58	9.08	4.70
	*p*	<0.001	0.004	0.036
	$\eta_{\text{p}}^{2}$	0.52	0.18	0.10
Reaction time	*F*_(1,41)_	286.57	0.48	9.22
	*p*	<0.001	0.49	0.004
	$\eta_{\text{p}}^{2}$	0.88	0.01	0.18
FN400	*F*_(1,41)_	7.21	91.23	35.92
	*p*	0.01	<0.001	<0.001
	$\eta_{\text{p}}^{2}$	0.15	0.69	0.05
SN	*F*_(1,41)_	20.41	6.64	6.45
	*p*	<0.001	0.01	0.02
	$\eta_{\text{p}}^{2}$	0.33	0.13	0.14

For “correct” response rates, the interaction between premise monotonicity and conclusion congruency reached statistical significance (Table 1). When the factor of premise monotonicity was entered into the *post hoc* analysis, the results suggested that T arguments featured higher “correct” response rates than S arguments under both congruent (T+: *M* = 0.97, *SD* = 0.04; S+: *M* = 0.93, *SD* = 0.06; *F*_(1,41)_ = 35.39, *p* < 0.001, $\eta_{\text{p}}^{2}$ = 0.46) and incongruent conditions (T−: *M* = 0.99, *SD* = 0.02; S−: *M* = 0.96, *SD* = 0.04; *F*_(1,41)_ = 13.44, *p* = 0.001, $\eta_{\text{p}}^{2}$ = 0.25).

For reaction times, the interaction between premise monotonicity and conclusion congruency was also significant (Table 1). When the factor of premise monotonicity was entered into the *post hoc* analysis, the results suggested that T arguments featured shorter reaction times than S arguments under both congruent (T+: *M* = 726, *SD* = 123; S+: *M* = 1,078, *SD* = 181; *F*_(1,24)_ = 359.96, *p* < 0.001, $\eta_{\text{p}}^{2}$ = 0.90) and incongruent conditions (T−: *M* = 743, *SD* = 126; S−: *M* = 1,041, *SD* = 207; *F*_(1,24)_ = 161.28, *p* < 0.001, $\eta_{\text{p}}^{2}$ = 0.80). For decision threshold, T arguments exhibited a more liberal decision threshold than S arguments (T: *M* = 0.07, *SD* = 0.22; S: *M* = 0.15, *SD* = 0.27; *t*_(41)_ = 2.01, *p* = 0.05, Cohen’s *d* = 0.31).

### ERP Results

**Figure 1:**
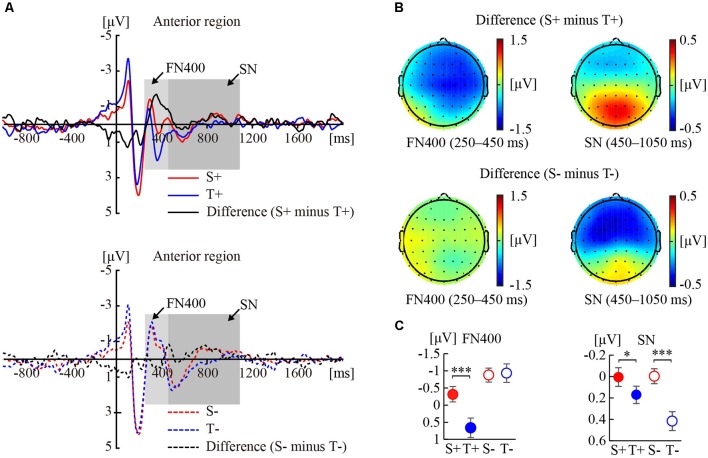
Event-related potential (ERP) responses to the premise monotonicity effect. **(A)** Grand-averaged waveforms elicited by S+ and T+ arguments and the difference waveform (S+ minus T+) in the anterior region (top); grand-averaged waveforms elicited by S− and T− arguments and the difference waveform (S− minus T−) in the anterior region (bottom). **(B)** Topographies of the difference waveforms of FN400 in the 250–450 ms interval and sustained negativity (SN) in the 450–1,050 ms interval. **(C)** Statistical comparisons of premise monotonicity (S, T) and conclusion congruency (+,−) in the average amplitudes of FN400 and SN. Error bars indicate the standard error of the mean (SEM). *p < 0.05; ***p < 0.001 for the comparison of means.

Figure 1 shows the ERP responses to the premise monotonicity and their topographies under congruent (Figure 1A) and incongruent (Figure 1B) conditions.

#### FN400

As shown in Table 1, the interaction between premise monotonicity and conclusion congruency was significant. When the factor of premise monotonicity was entered into the *post hoc* analysis, the results suggested that S arguments elicited larger FN400 amplitudes than T arguments under congruent conditions (*F*_(1,41)_ = 26.34, *p* < 0.001, $\eta_{\text{p}}^{2}$ = 0.39), but elicited amplitudes similar to those of T arguments under incongruent conditions (*F*_(1,41)_ = 0.08, *p* = 0.77, $\eta_{\text{p}}^{2}$ = 0.002).

#### Sustained Negativity (SN)

As shown in Table 1, the interaction between premise monotonicity and conclusion congruency was significant. When the factor of premise monotonicity was entered into the *post hoc* analysis, the results suggested that S arguments elicited larger SN than T arguments under both congruent (*F*_(1,41)_ = 4.09, *p* = 0.049, $\eta_{\text{p}}^{2}$ = 0.09), and incongruent conditions (*F*_(1,41)_ = 26.70, *p* < 0.001, $\eta_{\text{p}}^{2}$ = 0.39).

### Time-Frequency Domain Results

**Figure 2:**
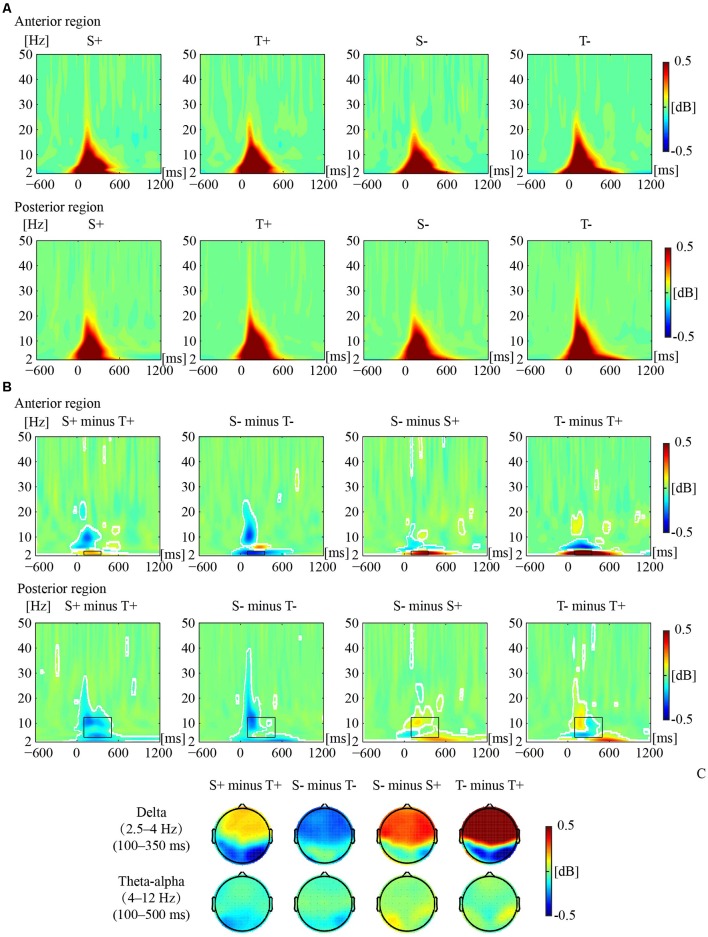
Phase-locked electrophysiological (EEG) power responses to the premise monotonicity effect. **(A)** Grand-averaged power evoked by S+, T+, S−, and T− arguments in the anterior and posterior regions. **(B)** The EEG power of “S+ minus T+”, “S− minus T−”, “S− minus S+”, and “T− minus T+” is shown in the anterior and posterior regions. White outlines indicate significant [*p* < 0.01, false discovery rate (FDR) corrected] time–frequency pixels in the bootstrapping statistical analysis. Black outlines indicate time-frequency regions of interest (TF-ROIs) of the delta (2.5–4 Hz) and theta-alpha (4–12 Hz) bands. **(C)** Power difference topographies of the delta and theta-alpha bands.

**Figure 3:**
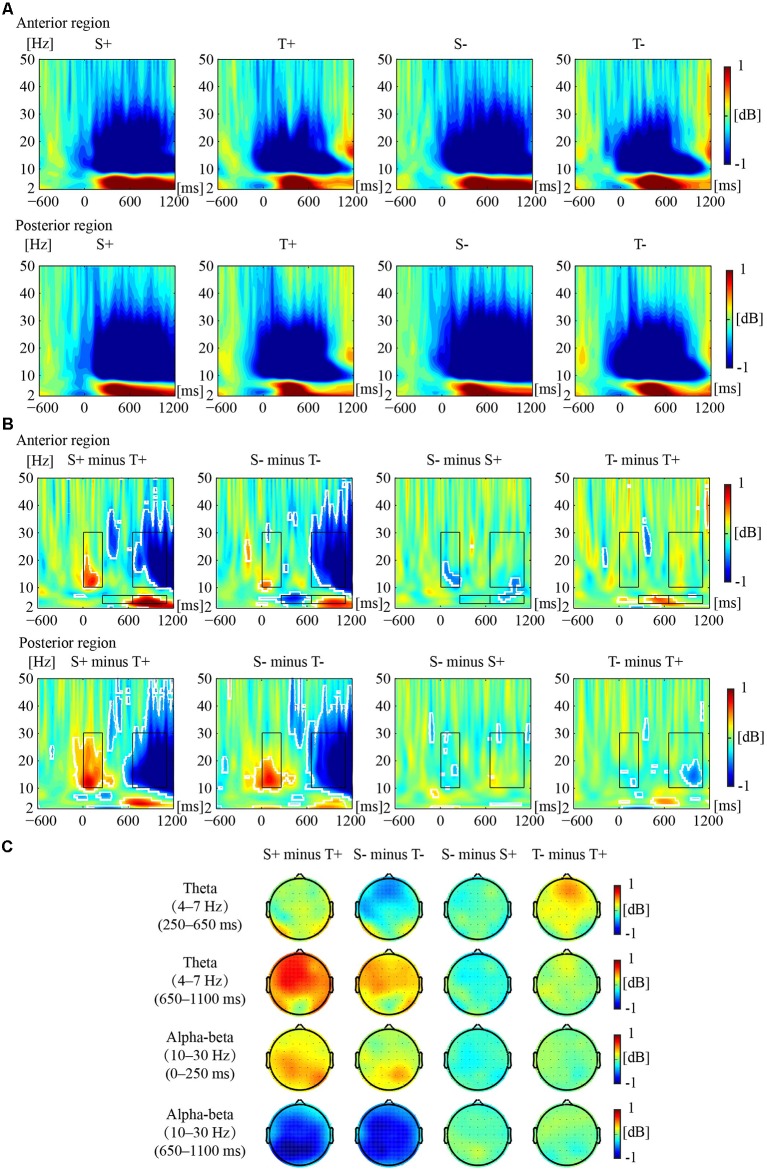
Non-phased-locked EEG power responses to the premise monotonicity effect. **(A)** Grand-averaged power produced by S+, T+, S−, and T− arguments in the anterior and posterior regions. **(B)** The EEG power of “S+ minus T+”, “S− minus T−”, “S− minus S+”, and “T− minus T+” is shown in the anterior and posterior regions. White outlines indicate the significant (*p* < 0.01, FDR corrected) time–frequency pixels in the bootstrapping statistical analysis. Black outlines indicate TF-ROIs of the theta and the alpha-beta (10–30 Hz) bands. **(C)** Power difference topographies of the theta (250–650 ms and 650–1,100 ms) and alpha-beta (0–250 ms and 650–1,100 ms) bands.

Figure 2 illustrates the phase-locked EEG power and the corresponding topographies. Figure 3 illustrates the non-phased-locked EEG power and the corresponding topographies. Table 2 shows the results of the three-way repeated-measures ANOVAs of phase-locked and non-phase-locked EEG power.

**Table 2:** Results of the three-way repeated-measures ANOVAs of phase-locked and non-phase-locked power.

		Premise (P)	Conclusion (C)	Region (R)	P*C	P*R	C*R	P*C*R
Delta	*F*_(1,41)_	3.27	25.35	13.71	3.94	1.35	34.39	15.58
2.5–4 Hz	*p*	0.08	<0.001	0.001	0.05	0.25	<0.001	<0.001
100–350 ms	$\eta_{\text{p}}^{2}$	0.07	0.38	0.25	0.09	0.05	0.46	0.27
Theta-alpha	*F*_(1,41)_	14.07	0.02	2.07	0.90	4.60	0.80	1.64
4–12 Hz	*p*	0.001	0.88	0.16	0.35	0.04	0.38	0.21
100–500 ms	$\eta_{\text{p}}^{2}$	0.26	0.001	0.05	0.02	0.10	0.02	0.04
Theta	*F*_(1,41)_	1.53	1.87	15.83	7.00	2.92	0.19	1.98
4–7 Hz	*p*	0.22	0.18	<0.001	0.01	0.10	0.67	0.17
250–650 ms	$\eta_{\text{p}}^{2}$	0.04	0.04	0.28	0.15	0.07	0.01	0.05
Theta	*F*_(1,41)_	15.22	0.56	31.49	0.01	5.53	3.58	0.70
4–7 Hz	*p*	<0.001	0.46	<0.001	0.92	0.02	0.07	0.41
650–1,100 ms	$\eta_{\text{p}}^{2}$	0.27	0.01	0.43	<0.001	0.12	0.08	0.02
Alpha-beta	*F*_(1,41)_	23.13	7.08	11.89	3.61	9.05	0.23	0.40
10–30 Hz	*p*	<0.001	0.01	0.001	0.07	0.004	0.64	0.53
0–250 ms	$\eta_{\text{p}}^{2}$	0.36	0.15	0.23	0.08	0.18	0.01	0.01
Alpha-beta	*F*_(1,41)_	105.14	0.56	1.90	<0.001	4.27	0.24	7.22
10–30 Hz	*p*	<0.001	0.46	<0.001	0.99	0.05	0.63	0.01
650–1,100 ms	$\eta_{\text{p}}^{2}$	0.72	0.01	0.32	<0.001	0.09	0.01	0.15

#### The Results on Phase-Locked EEG Power

For delta band power, the interaction among premise monotonicity, conclusion congruency, and brain region was significant (Table 2). Thus, we performed *post hoc* repeated-measures ANOVAs with premise monotonicity and conclusion congruency as within factors in the anterior and posterior region. In the anterior region, the interaction between premise monotonicity and conclusion congruency was significant (*F*_(1,41)_ = 10.178, *p* = 0.002, $\eta_{\text{p}}^{2}$ = 0.21). When the factor of premise monotonicity was entered into the *post hoc* analysis, the results suggested that S+ evoked larger delta power than T+ (*F*_(1,41)_ = 4.39, *p* = 0.04, $\eta_{\text{p}}^{2}$ = 0.10), while S− evoked smaller delta power than T− (*F*_(1,41)_ = 6.28, *p* = 0.02, $\eta_{\text{p}}^{2}$ = 0.13). When the factor of conclusion congruency was entered into the *post hoc* analysis, the results showed that incongruent conditions elicited larger delta power than congruent conditions under both S (*F*_(1,41)_ = 22.11, *p* < 0.001, $\eta_{\text{p}}^{2}$ = 0.35) and T arguments (*F*_(1,41)_ = 40.24, *p* < 0.001, $\eta_{\text{p}}^{2}$ = 0.50). In the posterior region, the interaction between premise monotonicity and conclusion congruency was not significant (*F*_(1,41)_ = 0.42, *p* = 0.52, $\eta_{\text{p}}^{2}$ = 0.01), nor was the main effect of conclusion congruency (*F*_(1,41)_ = 1.05, *p* = 0.31, $\eta_{\text{p}}^{2}$ = 0.03). However, the main effect of premise monotonicity was significant (*F*_(1,41)_ = 9.58, *p* = 0.004, $\eta_{\text{p}}^{2}$ = 0.19), T arguments evoking larger delta power than S arguments.

Concerning theta-alpha band power, no significant interaction was found among premise monotonicity, conclusion congruency, and brain region (Table 2). Among all the two-way interaction effects, only the interaction between premise monotonicity and brain region was significant. When the factor of premise monotonicity was entered into the *post hoc* analysis, the results showed that T arguments elicited larger theta-alpha power than S arguments at posterior (*F*_(1,41)_ = 18.23, *p* < 0.001, $\eta_{\text{p}}^{2}$ = 0.31), but not anterior regions (*F*_(1,41)_ = 3.71, *p* = 0.06, $\eta_{\text{p}}^{2}$ = 0.08).

#### The Results on Non-phase-Locked EEG Power

For early theta band power, as shown in Table 2, no significant interaction was found among premise monotonicity, conclusion congruency, and brain region. However, the interaction between premise monotonicity and conclusion congruency was significant. When the factor of premise monotonicity was entered into the *post hoc* analysis, the results suggested that T+ induced theta power similar to S+ (*F*_(1,41)_ = 0.14, *p* = 0.71, $\eta_{\text{p}}^{2}$ = 0.004), while T− induced larger theta power than S− (*F*_(1,41)_ = 5.37, *p* = 0.03, $\eta_{\text{p}}^{2}$ = 0.12). When the factor of conclusion congruency was entered into the *post hoc* analysis, the results suggested that T− induced larger theta power than T+ (*F*_(1,41)_ = 6.58, *p* = 0.01, $\eta_{\text{p}}^{2}$ = 0.14), while S+ and S− induced similar theta power (*F*_(1,41)_ = 0.85, *p* = 0.36, $\eta_{\text{p}}^{2}$ = 0.02).

For late theta band power, no significant interaction was found among premise monotonicity, conclusion congruency, and brain region (Table 2). However, the interaction between premise monotonicity and brain region was significant. When the factor of premise monotonicity was entered into the *post hoc* analysis, the results showed that S arguments induced larger theta power than T arguments in both anterior (*F*_(1,41)_ = 15.81, *p* < 0.001, $\eta_{\text{p}}^{2}$ = 0.28) and posterior regions (*F*_(1,41)_ = 9.24, *p* = 0.004, $\eta_{\text{p}}^{2}$ = 0.18).

For early alpha-beta band power, as shown in Table 2, no significant interaction was found among premise monotonicity, conclusion congruency, and brain region. However, the interaction between premise monotonicity and brain region was significant. When the factor of premise monotonicity was entered into the *post hoc* analysis, the results showed that S arguments elicited larger alpha-beta power than T arguments in both anterior (*F*_(1,41)_ = 4.56, *p* = 0.04, $\eta_{\text{p}}^{2}$ = 0.10) and posterior regions (*F*_(1,41)_ = 41.73, *p* < 0.001, $\eta_{\text{p}}^{2}$ = 0.50).

For late alpha-beta band power, the interaction among premise monotonicity, conclusion congruency, and brain region was significant (Table 2). Thus, *post hoc* repeated-measures ANOVAs were performed in the anterior and posterior regions. The main effects of premise monotonicity were significant in both the anterior (*F*_(1,41)_ = 82.97, *p* < 0.001, $\eta_{\text{p}}^{2}$ = 0.67) and posterior regions (*F*_(1,41)_ = 87.61, *p* < 0.001, $\eta_{\text{p}}^{2}$ = 0.68), with T arguments producing larger alpha-beta power than S arguments.

### The Correlation Between Behavioral Response and Electrophysiology Parameters

To further analyze the relationship between variations of behavioral responses and changes in the electrophysiology parameters, Spearman’s correlation analysis was performed. The variations of behavioral responses (“correct” response rates, response strength, and decision threshold *c*) and electrophysiology parameters (FN400 amplitudes, SN amplitudes, phase-locked anterior delta power, phase-locked posterior theta-alpha power, non-phase-locked anterior theta power, non-phase-locked posterior alpha-beta power) were computed as S minus T under congruent and incongruent conclusion conditions. Only significant correlations are reported in the following paragraphs.

Under congruent conditions, the results suggested that the variation in “correct” response rates was significant correlated with that in FN400 amplitudes [*r* = −0.37, *p* = 0.02, 95%CI (−0.61, −0.08)], and with the variation in phase-locked anterior delta power [*r* = 0.31, *p* = 0.05, 95%CI (0.001, 0.56)]. The results also suggested that the variation in decision threshold *c* was significant correlated with that in FN400 amplitudes [*r* = 0.31, *p* = 0.05, 95%CI (0.01, 0.56)], and with that in phase-locked posterior theta-alpha power [*r* = −0.41, *p* = 0.01, 95%CI (−0.63, −0.12)].

Under incongruent conditions, the results suggested that the variation in response strength significantly correlated with the variation in non-phase-locked anterior early theta power [*r* = 0.31, *p* = 0.05, 95%CI (0.004, 0.56)]. The results also suggested that the variation in “correct” response rates was significantly correlated with that in phase-locked posterior theta-alpha band [*r* = −0.34, *p* = 0.03, 95%CI (−0.58, −0.04)], and with that in non-phase-locked anterior late theta power [*r* = 0.31, *p* = 0.05, 95%CI (0.01, 0.56)].

## Discussion

Cui et al. (2018) explored the dependence of ERP responses to inductive decisions on the premise monotonicity effect during category-based induction. However, the neural oscillation profiles of this effect, which can provide insight into the cognitive processing that lost in traditional ERP analysis, were not investigated in their study. In this study, we investigated the EEG oscillatory activity related to the premise monotonicity effect during category-based induction, using time-frequency analysis techniques. The data suggested that the premise monotonicity effect was revealed by response strength, “correct” response rate, reaction times, decision threshold, FN400 amplitudes, and SN amplitudes. Moreover, the time-frequency analysis showed that the premise monotonicity effect was revealed by delta and theta-alpha in phase-locked EEG power, and theta and alpha-beta in non-phase-locked EEG power.

In the present study, the premise monotonicity effect during category-based induction was revealed by response strength, “correct” response rates, reaction times, and decision threshold, in agreement with previous studies (Osherson et al., 1990; Feeney, 2007; Cui et al., 2018; Hayes and Heit, 2018). Moreover, the premise monotonicity effect during category-based induction was revealed by FN400 and SN amplitudes, similarly to what reported by Cui et al. (2018), with different high-pass filter parameters.

In phase-locked power, the premise monotonicity effect during semantic category-based induction was revealed by delta power in the 100–350 ms time window. In the cognitive domain, delta oscillations are related to signal detection, decision-making, attention, inhibition, or working memory (Spironelli and Angrilli, 2010; Putman, 2011; Leszczyński et al., 2015; Rac-Lubashevsky and Kessler, 2018; reviews, Knyazev, 2012; Harmony, 2013; Güntekin and Başar, 2016).

We speculate that working memory (WM) updating during category-based induction modulates the phase-locked anterior delta power. In the present study, compared with congruent conclusions, incongruent conclusions involve a categorical membership inconsistent between premises and conclusions, which generates more information to be updated in the WM. Moreover, T− needs more WM to be updated than S−. This is because each T− argument includes two premise categories unrelated to the conclusion category, whereas S− only includes one. As a result, T− involves increased conceptual confliction than S−, producing an increased WM updating requirement. In addition, T+ needs less WM to be updated than S+. This is because T+ has more premise categories than S+, which provide more evidence to support induction. As a result, T+ involves increased conceptual fluency than S+, leading to a decreased WM updating requirement. Therefore, incongruent conclusions generate more information to be updated in the WM, producing larger anterior delta power than congruent conclusions; T− needs more WM to be updated than S−, producing larger anterior delta power; and T+ needs less WM to be updated than S+, producing less anterior delta power. These speculations are supported by Rac-Lubashevsky and Kessler (2018), who suggested that WM updating is related to an increase in anterior delta power.

In phase-locked power, the premise monotonicity effect during semantic category-based induction was also revealed by the posterior theta-alpha power. We hypothesize that the facilitation of inference-driven processing and subjectively perceived inductive confidence produce the variations of the posterior theta-alpha power. In the present study, T arguments elicited more definite responses than S arguments, suggesting that inference-driven processing is facilitated by T arguments. This facilitated processing may occur *via* the inhibition of specific stimulus-based bottom-up processing demands during inferences, producing larger phase-locked posterior alpha power. This hypothesis is in line with previous studies which suggested that facilitating task performance *via* preventing the reorientation of stimulus-driven irrelevant stimulations is associated with increased posterior alpha power (e.g., Benedek et al., 2014; Fink et al., 2018; a review, Klimesch, 2012). Moreover, the facilitation of inference-driven processing of T arguments led to stronger subjectively perceived inductive confidence than S arguments; as a result, T arguments produced larger posterior theta power than S arguments. This hypothesis is supported by Wynn et al. (2019), who suggested that stronger subjective perceived confidence is related to an increase in posterior theta power. In a recognition task, they found that evoked posterior theta power was stronger during high-confidence than low-confidence responses in the retrieval phase, suggesting that increased evoked theta power indicates stronger subjectively perceived confidence.

In non-phase-locked power, the premise monotonicity effect during semantic category-based induction was revealed by the anterior theta power. Specifically, in the present study, T− induced larger theta power than S− in a relatively early time window (250–650 ms), while S arguments induced larger theta power than T arguments in a relatively late time window (650–1,100 ms). We hypothesize that top-down guided cognitive control requirement and adaptive control generated the increased anterior theta power. Specifically, incongruent conclusions involve a confliction of categorical membership, which leads to prediction errors. T− arguments involve two violations of categorical membership, while S− arguments only involve one. Hence, T− arguments need more cognitive control than S−, producing larger theta power in a relatively early time window. On the other hand, under congruent conclusions, no conflictions occur. As a result, T+ and S+ produced similar theta power in a relatively early time window. This hypothesis is in line with previous studies which indicated that the need for cognitive control is related to an increased early anterior theta power (Cavanagh et al., 2010; van de Vijver et al., 2011; Rommers et al., 2017; Rac-Lubashevsky and Kessler, 2018; Cooper et al., 2019). For example, Rommers et al. (2017) found that compared with sentences which included an expected final word, theta band power was enhanced in those that included an unexpected final word. They suggested that the need for cognitive control was increased by the prediction error in the latter condition, generating increased theta power.

On the other hand, we speculate that implementing adaptive control is associated with the relatively late anterior theta power. In the present study, since the premise sample size was smaller in S than in T arguments, the former involved more decision uncertainty than the latter. Moreover, S arguments led to longer reaction times and more conservative decision thresholds than T arguments, suggesting increased response caution. As a result, S arguments involve more adaptive control, producing larger anterior theta power than T arguments in a relatively late time window. This is supported by previous studies which suggested that increased decision uncertainty and response caution led to implementing adaptive control and that implementing adaptive control is associated with anterior theta power (Cavanagh et al., 2012; Cavanagh and Shackman, 2015; Zavala et al., 2016). For instance, Zavala et al. (2016) found that in a dot motion discrimination task with manipulated levels of uncertainty, trials with increased levels of uncertainty generated larger anterior theta power, suggesting that larger anterior theta power was related to increased adaptive cognitive control.

In non-phase-locked power, the premise monotonicity effect during semantic category-based induction was revealed by the alpha-beta band power. Specifically, T arguments induced smaller alpha-beta power than S arguments in the 0–250 ms time window, but larger alpha-beta power than S arguments in the 650–1,100 ms time window. We speculate that the unification of the lexical elements for sentence comprehension modulates the alpha-beta power in the relatively early time window (0–250 ms), while the unification of inference-driven information modulates it in the relatively late time window (650–1,100 ms). In the present study, T arguments have larger premise sample size than S arguments. Therefore, the unification of premises and conclusion for semantic comprehension in the T condition was more complex than in the S condition, producing smaller alpha-beta power in T arguments than in S arguments. Moreover, in the present study, compared with T arguments, S arguments have relatively weaker evidence to make an inductive decision, and thus lead to more complex inference-driven information integration and interpretive processes (Cui et al., 2018). Therefore, T arguments produced larger alpha-beta power than S arguments in the 650–1,100 ms time window. These hypotheses are based on previous studies which suggested that the unification processing is related to alpha-beta power, involving the unification of the lexical elements for sentence comprehension (Davidson and Indefrey, 2007; Bastiaansen et al., 2008; Hagoort, 2013; Lam et al., 2016; Drijvers et al., 2018), and the unification of high level cognitive operations *via* reprocessing or reanalysis of the sentences (Palva and Palva, 2007; Spironelli and Angrilli, 2010; Kielar et al., 2014).

The neural oscillation profiles revealed in the premise monotonicity effect during semantic category-based induction provide evidence to support the connectionist models of semantic cognition (Rogers and McClelland, 2008, 2014). These models hypothesize that cognitive processing units during semantic cognition are organized in hierarchical layers, which can include stimuli-driven layers and experience-based layers, and can influence each other, with a parallel distribution. In the present study, the premise monotonicity effect during semantic category-based induction was revealed by phased-locked and non-phased locked EEG power, indexing stimuli-driven bottom-up processes and experience-based top-down processes, supporting the view that cognitive processing units during semantic tasks are organized in various layers. Moreover, in the present study, various neural oscillation bands were generated in overlapping time windows, suggesting parallel cognitive processing units. For example, both non-phase-locked theta and non-phase-locked alpha-beta power were generated in the 650–1,100 ms time window. Activity in various neural oscillation bands is associated with distinctive cognitive processing, and is thus generated in the same time window, suggesting that the cognitive processes related to the activity of distinctive neural oscillation bands take place in parallel, as the connectionist models of semantic cognition hypothesized.

A potential limitation of the present study is that only one and two exemplars were used to manipulate the premise monotonicity effect during semantic category-based induction, which may lead to potential confounding factors. However, the data suggest that under congruent conditions, the variations of the “correct” response rates were significantly correlated with FN400 amplitudes and phase-locked anterior delta power, while the variations of decision threshold were significantly correlated with FN400 amplitudes and phase-locked posterior theta-alpha power. These results suggest co-variation between brain responses and behavior responses, providing evidence that potential confounding factors for the premise monotonicity effect during semantic category-based induction were well controlled.

Further studies are required to elucidate the location in the brain where the neural oscillations for the premise monotonicity effect during category-based induction are generated. One of the limitations of the EEG technique is the poor spatial resolution in brain activity. Further studies can use magnetoencephalography (MEG) and functional magnetic resonance imaging (fMRI), which have good spatial resolution, to explore the spatial activity of the brain in the premise monotonicity effect during category-based induction.

In conclusion, the present findings contribute to the understanding of neural oscillation profiles of the premise monotonicity effect during semantic category-based induction. The results showed that the premise monotonicity effect during semantic category-based induction effect is revealed by both phase-locked and non-phase-locked power. In phase-locked power, T− evokes larger anterior delta power than S−, while S+ evokes larger delta power than T+, reflecting the variations in WM updating due to the premise monotonicity effect during category-based induction. Moreover, T arguments evoke larger posterior theta-alpha power than S, suggesting stronger subjectively perceived confidence. In non-phase-locked power, T− induced larger early theta power than other conditions between 250 and 650 ms, due to the increased need for cognitive control. Moreover, S arguments induced larger late theta power than T between 650 and 1,100 ms, suggesting that S arguments implement more adaptive control towards a cautious decision than T arguments. Furthermore, T arguments induce less alpha-beta power than S arguments in the 0–250 ms window, which is related to the unification of premise and conclusion for argument comprehension; whereas S arguments induced less alpha-beta power than T arguments in the 650–1,100 ms window, which is related to the unification of inference-driven information. Thus, the neural oscillation profiles of the premise monotonicity effect during semantic category-based induction were elucidated. Moreover, the connectionist models of category-based induction were supported by generating both phase-locked and non-phase locked power of premise monotonicity effect during semantic category-based induction, with the various bands of neural oscillation activity generated in overlapping time windows.

## Data Availability Statement

The datasets generated for this study are available on request to the corresponding author.

## Author Contributions

CL, MS and FX contributed to the conception and design of the study. FX contributed to the acquisition of data. MS and FX analyzed the data. CL and MS wrote the first draft of the manuscript. All authors contributed to the revision of the manuscript, read and approved the submitted version.

## Conflict of Interest

The authors declare that the research was conducted in the absence of any commercial or financial relationships that could be construed as a potential conflict of interest.
